# Immunomodulatory nano-preparations for rheumatoid arthritis

**DOI:** 10.1080/10717544.2022.2152136

**Published:** 2022-12-08

**Authors:** Chenglong Li, Yangyun Han, Xianjin Luo, Can Qian, Yang Li, Huaiyu Su, Guangshen Du

**Affiliations:** a Department of Pharmacy, The People’s Hospital of Deyang City, Deyang, P.R. China; b Department of Neurosurgery, The People’s Hospital of Deyang City, Deyang, P.R. China; c Pharmaceutical Biotechnology, Center for System-based Drug Research, Ludwig-Maximilians-Universität, Munich, Germany; d Key Laboratory of Drug Targeting and Drug Delivery Systems, Ministry of Education, West China School of Pharmacy, Sichuan University, Chengdu, P.R. China

**Keywords:** Immunomodulatory nano-preparations, rheumatoid arthritis, self-antigen, immunomodulator, antigen-specific immune tolerance

## Abstract

Rheumatoid arthritis (RA) is a systemic autoimmune disease (AD) caused by the aberrant attack of the immune system on its own joint tissues. Genetic and environmental factors are the main reasons of immune system impairment and high incidence of RA. Although there are medications on the market that lessen disease activity, there is no known cure for RA, and patients are at risk in varying degrees of systemic immunosuppression. By transporting (encapsulating or surface binding) RA-related self-antigens, nucleic acids, immunomodulators, or cytokines, tolerogenic nanoparticles—also known as immunomodulatory nano-preparations—have the potential to gently regulate local immune responses and ultimately induce antigen-specific immune tolerance. We review the recent advances in immunomodulatory nano-preparations for delivering self-antigen or self-antigen plus immunomodulator, simulating apoptotic cell avatars *in vivo*, acting as artificial antigen-presenting cells, and based on scaffolds and gels, to provide a reference for developing new immunotherapies for RA.

## 1. Introduction

As an organ- or systemic-specific inflammation, autoimmune disease (AD) is defined by the immune system’s failure to accurately discriminate between ‘self’ and ‘non-self’ (Y. Chen et al., 2020). According to epidemiological data, the incidence of 29 ADs is just under 10% worldwide (Cooper et al., 2009). With an incidence of 0.5%–1%, rheumatoid arthritis (RA) is one of the ADs with a high disability rate (Smolen et al., 2018). RA is a chronic innate and adaptive immunity mediated disease that primarily affects the peripheral joints (McInnes & Schett, 2011; Smolen et al., 2016). A variety of abnormally activated immune and non-immune cells, such as macrophages, fibroblast-like synoviocytes, and osteoclasts, collaborate to cause synovial inflammation and joint degeneration. The high level of rheumatoid factor (RF) and autoantibodies against cyclic citrullinated peptide or type II collagen (CII) *in vivo* are effective immunological indicators for the diagnosis of RA (Mullazehi et al., 2012; Gavrila et al., 2016). Functional disability is one of the main causes of the rise in costs for patients with difficult-to-treat RA (D2T RA), whose average yearly treatment costs are more than 27,000 euros (Roodenrijs et al., 2021). As a result, this illness has a significant impact on patients’ quality of life and places a significant social and financial burden on society.

Currently, the first- or second-line medications, including conventional synthetic disease-modifying anti-rheumatic drugs (DMARDs), targeted synthetic DMARDs, biological DMARDs, and glucocorticoids (GCs), function primarily by suppressing the immune system to varying degrees. These procedures inevitably raise the danger of infection and malignancy, and once the course of therapy is stopped, the autoimmune reaction will recur or become even more severe. In addition, a substantial percentage of patients with RA still show insufficient responses to those medications, such as 30% and 37% for methotrexate (MTX) and biologics, respectively (Moulis et al., 2018; Szostak et al., 2020). As an alternative therapy for immunosuppression, antigen-specific immune tolerance, a strategy based on the principle of recognizing the target antigen of autoimmune inflammatory attack and using it to stimulate the immune system to attenuate antigen-specific attack (Shakya & Nandakumar, 2018), means that the immune system retains the ability to respond to other antigens or injuries while lacking the destructive immune response to specific antigens (Serra & Santamaria, 2019). This idea was employed to treat allergy disorders over a century ago (Noon, 1911).

Helper T (Th) cell transformation from a proinflammatory to a regulatory or suppressive phenotype is the optimal therapeutic approach for RA. In contrast to conventional immunosuppressive medication, antigen-specific immune tolerance aims to reduce reactive immune cells or differentiate them into regulatory subtypes, including central and peripheral immune tolerance (Xing & Hogquist, 2012). Central tolerance refers to the export of CD4^+^CD25^+^FOXP3^+^ regulatory T (Treg) cells (Tregs) from the thymus to the periphery to exert regulatory function. Clonal deletion or clonal shift is the main mechanism controlling auto-reactive T cell in thymus, but those pathways cannot account for all autoimmune reactions. Consequently, healthy bodies also require peripheral immune tolerance to maintain immunological homeostasis. The ‘two signals’ approach is necessary for peripheral T cell activation (Baxter & Hodgkin, 2002). First, protein antigens are often broken down into peptides by antigen presenting cells (APCs), which are then shown as peptide–major histocompatibility complex (MHC)II complexes on the surface of APCs to bind to T cell receptor (TCR) and form the initial signal. In addition, APCs also need to upregulate costimulatory molecules including CD40, CD80, and CD86 as the second signal of T cell survival and proliferation. In contrast, T cells often lose their ability to exert effector activity and becoming ‘anergic’ when APCs engage with them in the absence of a second signal. The ‘anergic’ T cells could be either instructed to undergo ‘apoptosis’ or differentiate into Tregs to control and suppress other immune cells (Cifuentes-Rius et al., 2021). Therefore, the ‘dual signal’ combination of antigen transmission forms the basis of immune response, and it will be a useful strategy to treat RA and other ADs by altering this process to reestablish immunological tolerance to certain antigens.

Fortunately, tolerogenic dendritic cells (DCs) that were successfully produced *in vitro* have been utilized in clinical studies to restore the function of Tregs *in vivo*, and these trials originally showed good tolerance and safety (Benham et al., 2015; Bell et al., 2017). However, the clinical translation is prevented by the expense of collecting and expanding autologous cells, complicated technology, and individualization needs, and further research is required to determine its efficacy. Moreover, as an alternative to cell adoptive therapy, *in vivo* delivery of free antigens and immunomodulators also has limitations. For instance, it is challenging to accomplish by methodically administering free drugs the requirement that the antigen and immunomodulator be taken up by the same cell in order to induce the production of tolerogenic DC or other APCs *in vivo* (Jiang et al., 2018; Hong et al., 2020); transforming growth factor-β (TGF-β) can directly induce T cell to differentiate into Treg cell (W. Chen et al., 2003), but injection of free form is prone to off-target (McKarns & Schwartz, 2005). To increase safety and effectiveness, it is therefore vital to optimize the targeting and stability of the delivery approach. Immunomodulatory nano-preparations, which have the ability to target and stimulate the growth of tolerogenic APCs (APCs lacking co-stimulatory molecules or secondary signals) or Tregs as well as act as carriers for therapeutic molecules, could be used to achieve this. In this review, we will concentrate on the development of various immunomodulatory nano-preparations for the treatment of RA over the past few years, including fabrication techniques and mechanisms of action (Table 1). Since we are only discussing immunomodulatory nano-preparations based on disease-associated antigens, antigen-independent nanomedicines are not included in this work.

**Table 1.** Immunomodulatory nano-preparations for rheumatoid arthritis.

Carriers	Antigens	Immunomodulators	Routes of administration	Disease models	Mechanism of action	Result of action	Reference
PLGA, 300 nm	CII	NA	Oral	CIA (DBA/1)	Decrease the level of serum IgG anti-CII antibodies and CII-specific T cell proliferation	Prophylactic effect	(Kim et al., 2002)
PLGA, 333 nm	CII_256-270_	NA	Oral	CIA (DBA/1)	Induce productions of IL-4 and IL-10 in T cells of Peyer’s patches	Immune tolerance on DBA/1 mice	(Lee WK et al., 2005)
Liposome, 400 nm	methylated BSA	NF-κB inhibitors	Subcutaneous	Ag-induced inflammatory arthritis (C57BL/6)	Induction of Ag-specific Tregs; increase IL-10 production	Therapeutic effect	(Capini et al., 2009)
Liposome, 105/135 nm	aggrecan_89–103_	1α,25-dihydroxyvitamin D_3_	Subcutaneous	PGIA (BALB/cAnNCrl)	Decrease the frequencies of aggrecan-specific CD4^+^ T cells and PG-specific IgG	Prophylactic effect	(Galea et al., 2019)
Nanoemulsion, 130 nm	a citrullinated multiepitope self-antigen	Rapamycin	Intravenous	CIA; AIA (DBA/1, Balb/c)	Increase the frequencies of Tregs, Bregs and M2 macrophages; decrease TNF-α, IL-1β	Therapeutic effect	(Li et al., 2021)
Lipid-coated calcium phosphate NPs, 180 nm	a multiepitope citrullinated peptide	Rapamycin	Intravenous	CIA (Wistar rats)	Increase the rate of Tregs, IL-10; decrease the production of pro-inflammatory cytokines and antibody titers	Therapeutic effect	(X. Chen et al., 2021a)
Iron oxide NPs, <500 nm	mCII_259-273_ bound to MHCII	NA	Subcutaneous	CIA (HLA-DR4-IE-transgenic C57BL/10.M)	Induce the expansion of antigen-specific Tr1 cells; promote the differentiation of cognate B cells into Bregs	Therapeutic effect	(Clemente-Casares et al., 2016)
Hydrogel	Exosome derived from BMSCs	NA	Implanted in cartilage defect	Osteochondral defect models (SD rats)	Recruit BMSCs migration, inflation, proliferation; promote cartilage defect regeneration	Therapeutic effect	(Zhang FX et al., 2021)

PLGA, poly (lactic-*co*-glycolic acid); CII, type II collage; CIA, collagen-induced arthritis; BSA, bovine serum albumin; PGIA, proteoglycan-induced inflammatory arthritis; AIA, adjuvant induced arthritis; IL-10, interleukin-10; IL-1β, interleukin-1β; TNF-α, Tumor necrosis factor-α; BMSCs, bone marrow derived mesenchymal stem cells; Tr1, T-regulatory type 1; NA, not applicable.

## 2. *In vivo* distribution of immunoregulatory nano-preparations

APCs can selectively take up NPs in the circulatory system and then be filtered and enriched in peripheral immune organs like the lymph nodes and spleen (X. Chen et al., 2021b) owing to the body’s intrinsic capacity to actively trap virus-like particles (Kishimoto & Maldonado, 2018).

Synthetic particles with diameters ranging from 0.050 to 1 μm are easily phagocytosed by APCs, according to studies, and the biodistribution *in vivo* is greatly influenced by particle size and the method of administration (Getts et al., 2015). For instance, NPs administered subcutaneously, intramuscularly, or intradermally are more likely to interact with the lymphatic system than other routes, as shown by the following interactions: (1) Small particles (10–100 nm) typically migrate to lymph nodes through lymphatic capillaries (Jiang et al., 2017); (2) Larger particles (500–2000 nm) are typically transported to lymph nodes by APCs in the subcutaneous area (Manolova et al., 2008); and (3) Particles smaller than 10 nm can easily escape from lymph nodes (Jiang et al., 2017). Intravenously injected NPs were more likely to aggregate in the liver, spleen, lung, bone marrow, and some inflammatory organs. Particles smaller than 100 nm can evade the reticuloendothelial system’s (RES) clearance (Shao et al., 2015), while particles less than 5.5 nm are filtered by the kidney and eliminated in urine (Choi et al., 2007). Gut-associated lymphoid tissue (GALT) and nasopharynx-associated lymphoid tissue (NALT) are easily enriched in NPs delivered orally or through the nasal mucosa. Surface modifications with ligands or antibodies usually endowed the NPs with active targeting capabilities. For example, nano-preparations decorated with folate and dextran sulfate were utilized for targeting activated macrophage (Heo et al., 2014; Yang et al., 2017), surface coupling of CD11c antibody could improve the uptake efficiency of DCs (Stead et al., 2018), and CD3 antibody modification showed increased T cell targeting effect (Bahmani et al., 2018). In addition, positively charged nanoparticles had higher cytotoxicity but could be more preferentially internalized by APCs than negatively charged ones (Cifuentes-Rius et al., 2021).

## 3. Immunomodulatory nano-preparations for the delivery of self-antigens or self-antigens plus immunomodulators

**Figure 1.**
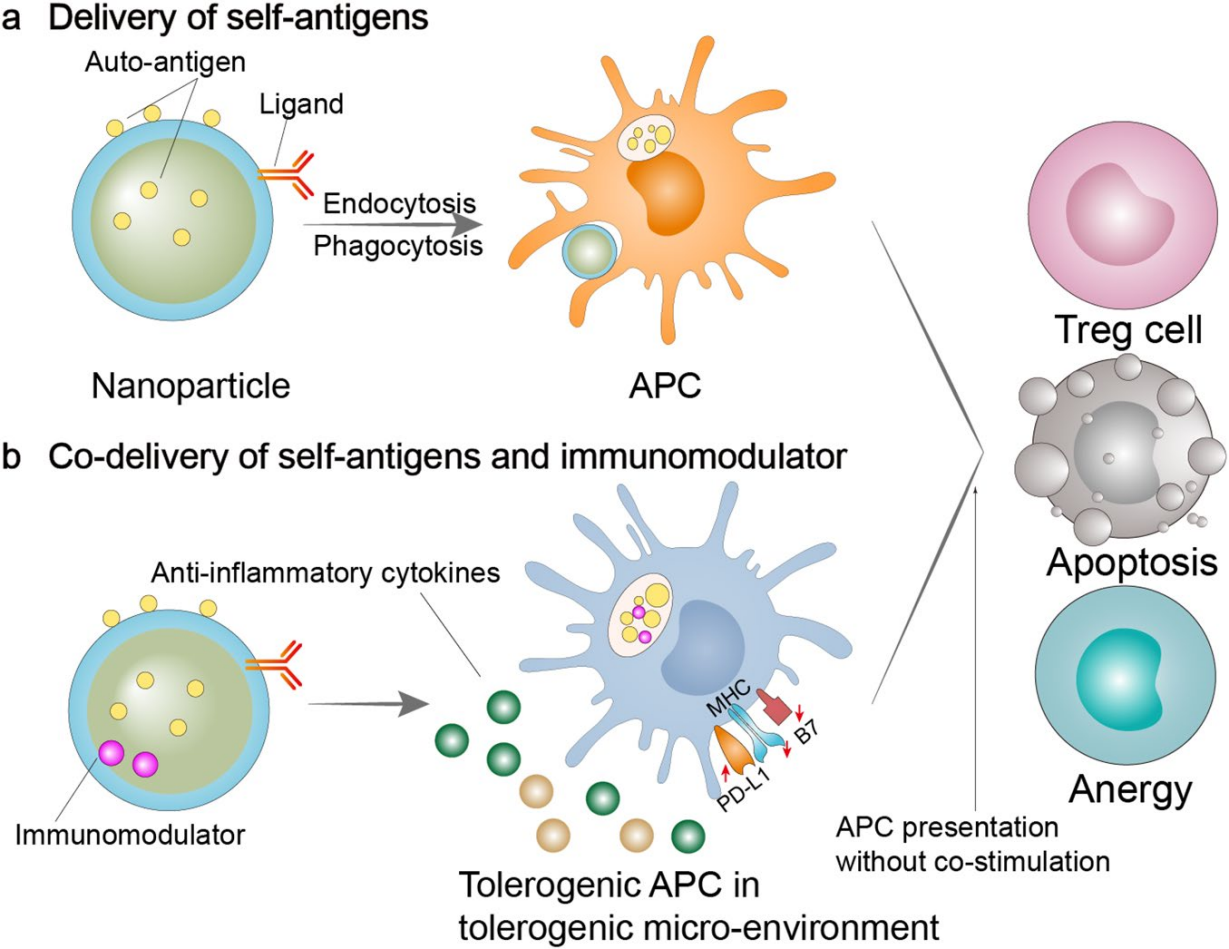
Immunomodulatory nano-preparations for the delivery of self-antigens or self-antigens plus immunomodulators. (a) NPs can be used for target delivery antigens to APCs via the surface attachment of ligands. These approaches can induce antigen presentation on APCs without co-stimulatory signals, leading to T cell anergy, apoptosis, or differentiate into a regulatory phenotype. (b) Co-delivering antigens and immunomodulators via NPs can result in antigen-specific immune tolerance. Drug-loaded NPs can be phagocytosed by APCs, and then releasing drugs intracellularly. Antigen presentation will be performed under the situation of high co-inhibitory molecule levels and low co-stimulatory molecule levels, thereby resulting in T cell anergy, apoptosis, or differentiate into a regulatory phenotype. APC, antigen-presenting cell; Treg cell, regulatory T cell; PD-L1, programmed cell death 1 ligand 1; MHC, major histocompatibility complex; B7, co-stimulatory molecule CD80 and CD86.

Immunomodulatory preparations can trigger the formation of tolerogenic APC after being taken up by APCs, and tolerogenic APC can promote T cell anergy, death, or differentiation into regulatory phenotype (Treg) (Figure 1). Therefore, the construction of NPs that can target and deliver antigens or antigens plus immunomodulators to APC may be an effective way to restore immune tolerance in RA.

### 3.1. Immunomodulatory nano-preparations for delivery of self-antigens

Poly(lactic-*co*-glycolic acid) (PLGA) is a biodegradable macromolecular polymeric pharmaceutical excipient approved by the Food and Drug Administration (FDA). The research of drug delivery frequently makes use of NPs made with PLGA due to their potent capacity to encapsulate hydrophobic small molecules, hydrophilic peptides, proteins, and other macromolecules, as well as their prolonged release effect (Mir et al., 2017). With just one oral dose, Kim et al.’s (2002) usage of PLGA NPs to encapsulate CII was able to drastically lessen the incidence and severity of collagen-induced arthritis (CIA) in mice. According to immunological data, lymph nodes from treated mice expressed more TGF-β and less tumor necrosis factor-α (TNF-α)-related messenger RNA (mRNA), which was accompanied by a significant drop in CII-specific IgG levels in their serum. W.K. Lee et al. (2005) prepared PLGA NPs to encapsulate the CII epitope peptides crosslinked with PEG (NP/PEG-pep), and administered intragastrically to DBA/1 mice on day 0. The results indicated that the percentage of interleukin-4^+^ (IL-4^+^) CD4^+^T cells and IL-10^+^CD4^+^ T cells in peyer’s patche (PP) of NP/PEG-pep immunized mice was significantly higher than that of the model or blank NPs group, and the level was comparable to that of mice immunized with CII protein for six times.

During the past few years, there have been significant advances in the technology used to pack mRNA into lipid nanoparticles (LNP) to cure or prevent disease (Samaridou et al., 2020; Hou et al., 2021). The primary disease-related self-antigens for multiple sclerosis (MS) are proteins derived from myelin, and experimental autoimmune encephalomyelitis (EAE) is a model for MS. To create a nanoparticle-formulated 1 methylpseudouridine-modified mRNA (M1 Ψ MRNA), an adjuvant-free mRNA LNP vaccine, Krienke et al. (2021) employed LNP to deliver mRNA that encodes EAE-related epitope antigen of myelin oligodendrocyte glycoprotein (MOG_35–55_). Following intravenous injection of M1ψ mRNA, the specific immunological response against MOG_35–55_ was successfully suppressed by increasing antigen-specific Tregs, decreasing MS-promoting Th1 cells, Th17 cells, and effector T cells. This approach dramatically decreased the occurrence and severity of MS in EAE without the systemic immunosuppression-related symptoms. The high flexibility and low cost of M1ψ mRNA technology will make it possible to design customized vaccination comprising various self-antigens to treat ADs.

Compared with immunoregulatory nano-preparation based on engineered biomaterials, the exosome-based nanomedicines have the advantages of low clearance rates and higher targeting (C. Li et al., 2022a). Casella et al. (2020) found that oligodendrocyte-derived extracellular vesicles (OL-EVs) are ∼240 nm and contain a range of myelin antigens. They injected OL-EVs intravenously into the EAE model induced by various myelin antigens, and successfully restored the immune tolerance by inducing immunosuppressive monocytogenesis and auto-reactive CD4^+^T cell apoptosis. Most importantly, the discovery that human oligodendrocytes may also release EVs, which include a number of self-antigens strongly related to MS, is crucial since it paves the way for the clinical translation of this technology. The significance of this study is that we still have the chance to treat MS in an antigen-specific manner without identifying the target antigens.

There is no question that immunomodulatory nano-preparation for delivery of self-antigens can induce antigen-specific immune tolerance. Nevertheless, the degree of tolerance may not be sufficient to overcome the robust autoimmunity and persistent inflammation in ADs, or even enhance the immune response.

### 3.2. Immunomodulatory nano-preparations for co-delivery of self-antigens and immunomodulators

Some small molecules, known as immunomodulators, have been shown to stimulate the generation of tolerogenic DC in an inflammatory microenvironment (Adorini et al., 2004; Svajger et al., 2010). However, when administered alone, it is simple to produce nonspecific systemic immunosuppression. The combination of self-antigen plus a small dosage of immunomodulator can improve the speed and extent of immunological tolerance to a certain antigen as well as lessen the shortcomings of the two alone. There are now two categories of immunomodulators that are frequently employed to construct immunomodulatory nano-preparations. The first is the nuclear factor kappa-B (NF-κB) signaling pathway inhibitors (Koide et al., 2015; Herrington et al., 2016; Riemann et al., 2017; L. Zhang et al., 2019; Barnabei et al., 2021), and the second is the mammalian target of rapamycin (mTOR) inhibitors (Benjamin et al., 2011; Chapman & Chi, 2014; Hoshii et al., 2014; Eskandari et al., 2022).

To effectively promote antigen-specific immunological tolerance and prevent the development of inflammation in RA models, Capini et al. (2009) employed liposomes to co-deliver antigen with various NF-κB inhibitors (including curcumin, quercetin, and Bay11-7082). After intravenous administration, drug-loaded liposomes were absorbed by MHCII^+^ APCs in peripheral immunological organs and caused the creation of tolerogenic APCs by blocking NF-κB signaling, which was followed by the development of antigen-specific Tregs.

**Figure 2.**
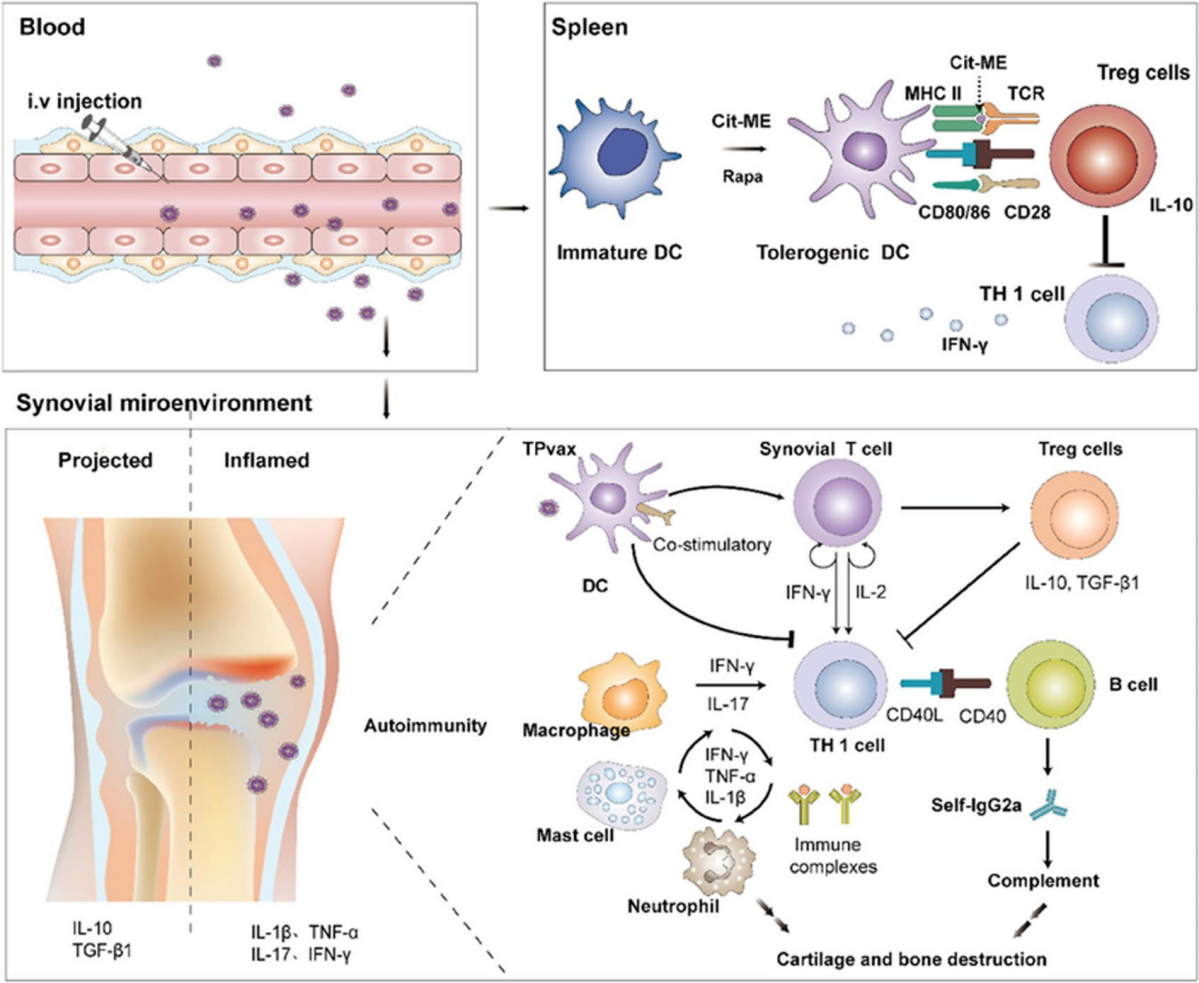
Schematic illustration of TPvax co-encapsulated with a multiepitope citrullinated peptide and rapamycin for anti-RA therapy by inducing antigen-specific immune tolerance. Reproduced with permission (X. Chen Xiaoyan et al., 2021a). Copyright © 2021 Elsevier Ltd.

Rapamycin, which was isolated from streptomyces hygroscopicus and was licensed by the FDA in 1999 to prevent kidney transplant rejection, inhibits the mTOR signaling pathway *in vivo* by binding to FK506-binding proteins. This compound decreases T cell proliferation and immune system function (Thomson et al., 2009), Fischer et al. (2009) also found it could increase the creation of tolerogenic DC *in vitro*. Selecta Biosciences researchers conducted a number of tests and discovered that NPs co-encapsulated with rapamycin and protein or peptide antigens could result in durable antigen-specific immune tolerance (Maldonado et al., 2015; Kishimoto & Maldonado, 2018; Sands et al., 2022). Immunomodulatory nano-preparations are injected subcutaneously, intravenously, or into lymph nodes, where they are preferentially taken up by APCs in the spleen, lymph nodes, and ectopic lymphoid structures (Maldonado et al., 2015; Tostanoski et al., 2016; Li et al., 2021). Subsequently, tolerogenic APC and Treg cell production are stimulated, and the generation of effector CD4^+^ and CD8^+^ cells is suppressed (Kishimoto et al., 2016). Therefore, co-loaded NPs could rectify the aberrant immune response mediated by effector T cells, especially in the treatment of EAE, and successfully restored the paralysis symptoms in the mouse model at the peak of the disease (Maldonado et al., 2015; Tostanoski et al., 2016). Furthermore, Tostanoski et al. (2016) successfully induced antigen-specific immune tolerance in EAE mice by injecting PLGA NPs co-loaded with MOG_35–55_ and rapamycin into lymph node, and permanently reversed the paralysis symptoms of the model. Recently, Sun and coworkers have made some headway in their exploration of the possibilities of this approach for the treatment of RA. To successfully induce immune tolerance in CIA and adjuvant-induced arthritis (AIA) mouse models, a novel nanoemulsion (NE) was created to co-deliver rapamycin and citrullinated multiepitope self-antigen to ectopic lymphoid structures at the inflamed joint (C. Li et al., 2021). The results showed that drug-loaded NEs have eventually halted the inflammatory progression of RA. They also developed a ‘tolerogenic polypeptide vaccine’ (TPvax) that was co-encapsulated with rapamycin and citrullinated peptide on the basis of the lipid-coated calcium phosphate nanoparticles. Following intravenous administration, CIA rats considerably increased their levels of Tregs and IL-10 while significantly decreasing the levels of inflammatory cytokines and auto-antibodies, which helped to restore the immunological balance (Figure 2) (X. Chen et al., 2021a).

Furthermore, other varieties of immunomodulators are also being studied (Szodoray et al., 2008; Charbonnier et al., 2015; Bivona et al., 2018; H.J. Lee et al., 2018; Brusko et al., 2020). The active form of vitamin D_3_ is 1,25-dihydroxyvitamin D_3_ (calcitriol), which can considerably raise the levels of the anti-inflammatory markers CD39 and CD73 on CD4^+^ T cells and boost TGF-β activity (Mann et al., 2015). The University of Queensland’s Galea et al. (2019) constructed liposomes co-loaded with an arthritogenic epitope of proteoglycan (Aggrecan_89-103_) and calcitriol. The outcomes of animal experiments showed that subcutaneously injected liposomes dramatically decreased the incidence and severity of proteoglycan-induced RA mice by preventing the growth and functionality of effector T cells and encouraging the generation of antigen-specific Tregs.

The dose of the immunomodulators, which determines whether the therapeutic effect is created by immunological tolerance or immunosuppression, is the crucial component of the above strategy.

## 4. Tolerogenic artificial antigen-presenting cells

Tolerogenic artificial APC that resembles the APC *in vivo* may have superior T cell targeting while immunomodulatory nano-preparation packs with self-antigen or self-antigen mixed with immunomodulator may have off-target effects. Nano-carriers unload ‘cargo’ without reaching the lesions or passively target non-target cells, often resulting in off-target toxicity or no therapeutic effect. Another subpopulation of CD4^+^ T-derived regulatory T cells, CD4^+^CD25^–^FOXP3^–^ T-regulatory type 1 (Tr1) cells have the ability to secrete the anti-inflammatory cytokines IL-10 and IL-21, and also express surface markers CD49b, LAG-3, and transcription factor c-Maf (Gagliani et al., 2013). NPs coated with ADs-associated antigenic peptide-MHC (pMHC) complex can attenuate ADs by reprogramming homologous effector T cells into disease-inhibiting Tr1 cells and undergoing massive expansion (Wraith, 2016). On the foundation of the aforementioned concept, Parvus Therapeutics, a biopharmaceutical company dedicated to developing specific immunomodulatory drugs for ADs, developed a Navicims platform technology for the treatment of MS, type 1 diabetes (T_1_D), RA, and other ADs. The core of Navacims, also known as peptides coupled to major histocompatibility complex class nanoparticles (pMHC-NPs), is a nanoscale iron oxide that is covered with polymer linked to the pMHC complex. By harboring diverse self-antigens, pMHC-NPs can produce targeted pharmacological actions against a variety of ADs (Clemente-Casares et al., 2016).

**Figure 3.**
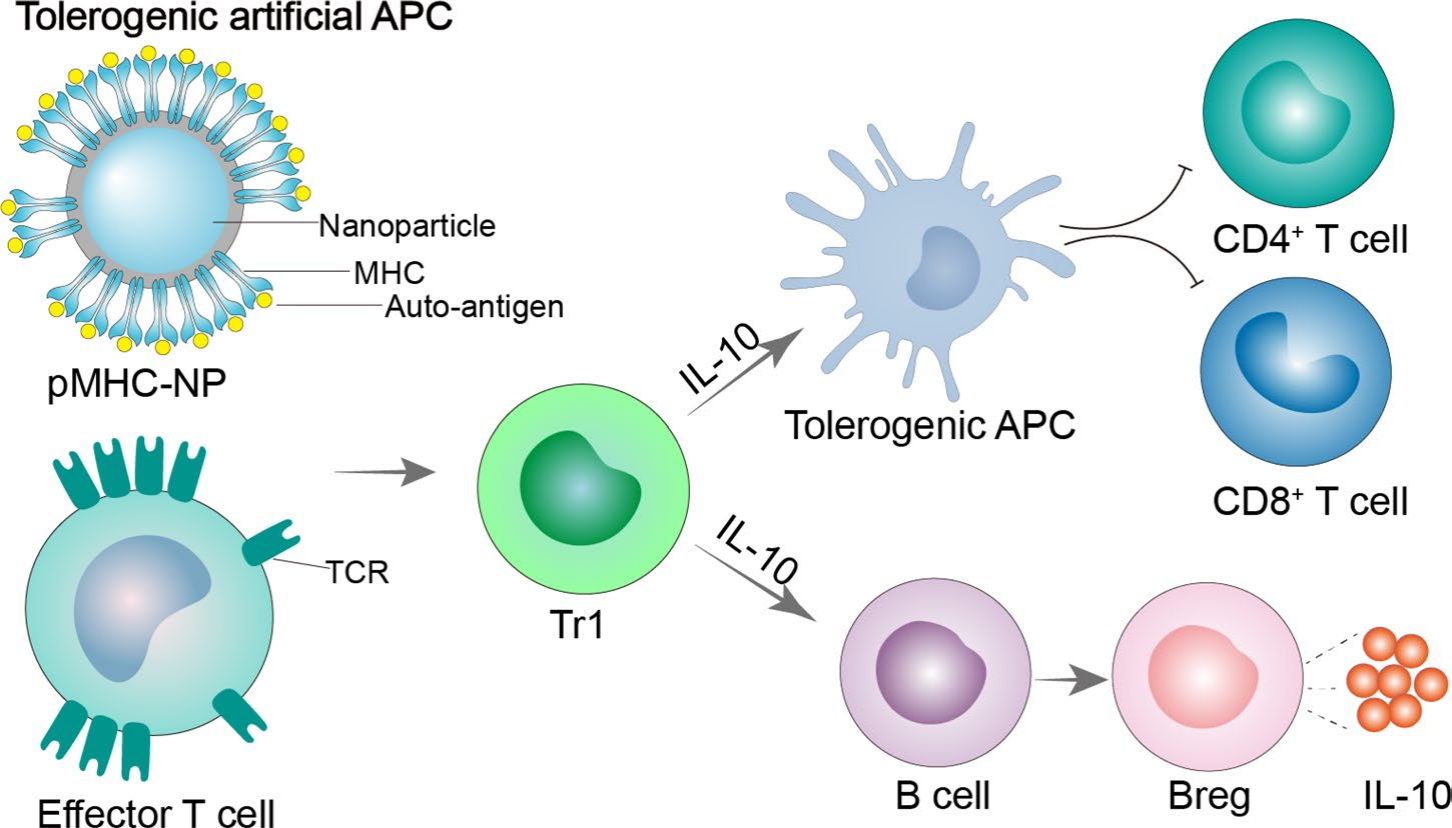
Tolerogenic artificial APC. In the absence of co-stimulation, artificial APCs display antigen on MHCI or MHCII to target CD8^+^ or CD4^+^ T cells, respectively. Artificial APCs have the ability to target T cells that are specific to an antigen, causing T cell anergy, apoptosis, or the generation of Tregs and Bregs. pMHC-NP, peptides coupled to major histocompatibility complex class nanoparticles; MHC, major histocompatibility complex; TCR, T-cell receptor; Tr1, Type 1 regulatory T cell; IL-10, interleukin-10.

Clemente-Casares et al. (2016) constructed the pMHCII-NPs carrying various disease-related antigens to treat RA and EAE mouse models, respectively. The results showed that pMHCII-NPs could effectively trigger the generation and proliferation of antigen-specific Tr1 cells, inhibit self-antigen-carried APCs, and promote the differentiation of B cells into Bregs without damaging the immune system (Figure 3). Furthermore, whereas pMHCII-NPs carrying CII epitope peptide could reduce RA, they had little impact on EAE. In contrast, pMHCII-NPs loaded with myelin antigen were able to suppress EAE but not RA. These findings demonstrated that the immunomodulatory effect of pMHCII-NPs was disease- and antigen-specific. In addition, the practical application of this approach was strengthened by the discovery that pMHCII-NPs could promote the differentiation and growth of human Tr1 cells in immunodeficient mice that had received transplants of human T and B cells. The same effect was observed in other ADs such as primary biliary cholangitis and primary sclerosis cholangitis (Umeshappa et al., 2019).

Tolerogenic artificial APC had an impact on other immune cells in addition to controlling the CD4^+^T cells differentiation, which in turn controlled the anti-autoimmune response. Tsai et al. (2010) found that pMHCI-NPs carrying T_1_D-related antigen suppressed autoreactive CD8^+^ T cells on islets in an epitope-specific manner by expanding CD8^+^ T cell subtypes with regulatory functions, thereby slowing down the progression of T_1_D. The research by Umeschappa et al. (2021) indicated that Tr1 cells and Bregs induced by pMHC-NPs could recruit neutrophils to the liver, and reprogramed their transcriptome under the influence of IL-10, IL-35, TGF-β1, IL-21, and granulocyte colony-stimulating factor (G-CSF) to generate regulatory neutrophils to treat experimental autoimmune hepatitis (AIH).

The advantages of tolerogenic artificial APC are that it works only in the infected organism and is not restricted to certain epitopes. Theoretically, pMHC-NPs containing any ADs-associated antigens may serve as a disease- and antigen-specific vaccine to prevent polyclonal autoimmune reactions.

## 5. Immunomodulatory nano-preparations mimicking apoptotic cell avatars *in vivo*

**Figure 4.**
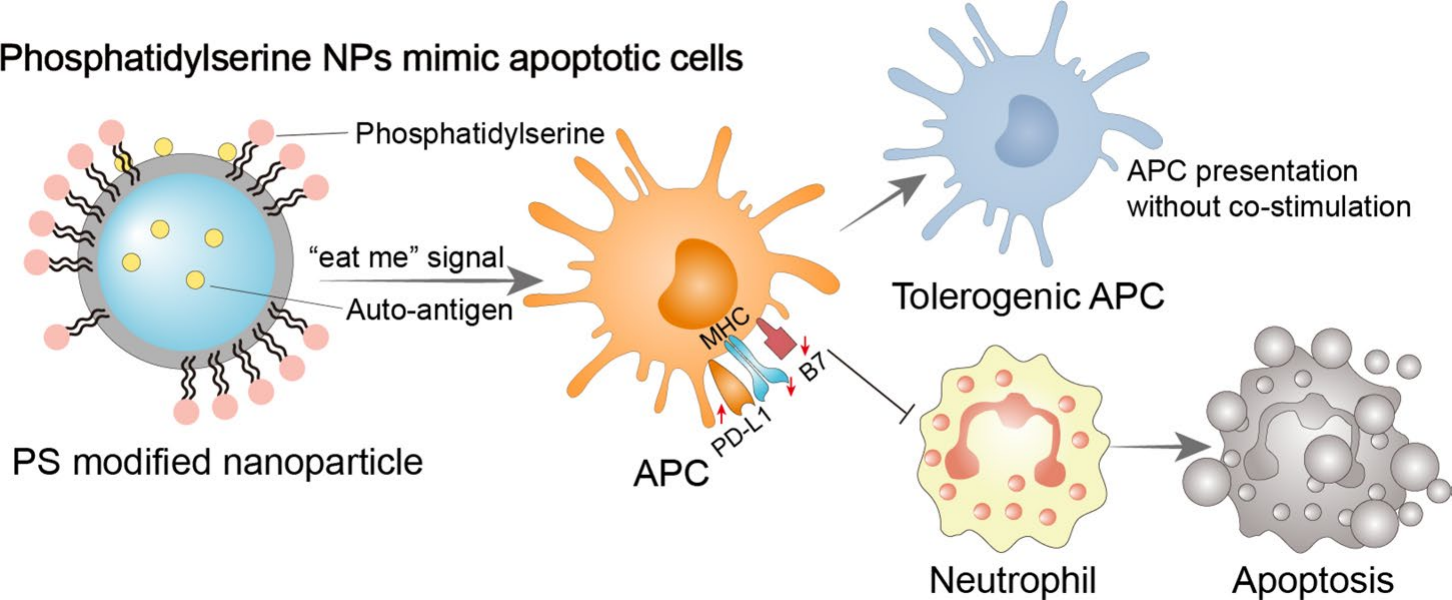
Immunomodulatory nano-preparations mimicking apoptotic cell avatars. Phosphatidylserine modified NPs send ‘eat me’ signal to APCs, and APCs differentiate into tolerogenic phenotypes while responding to antigen-loaded NPs. Tolerogenic APCs stop attracting neutrophils and perform immunomodulatory function. PS, phosphatidylserine; APC, antigen-presenting cell; Treg cell, regulatory T cell; PD-L1, programmed cell death 1 ligand 1; MHC, major histocompatibility complex; B7, co-stimulatory molecule CD80 and CD86.

Apoptosis is a process of programmed cell death that occurs in all tissues throughout the course of an individual’s life. After apoptosis has taken place, professional phagocytes eliminate dead cells through efferocytosis. Apoptosis is a crucial step to preserve homeostasis. Indeed, approximately one billion cells experience apoptosis each day in the human body without triggering an immune reaction or inflammation (Doran et al., 2020). Nevertheless, inadequate clearance of apoptotic cells may have harmful effects, including disruption of immunological homeostasis, a prolonged inflammatory response, and an intensified autoimmune response (Abdolmaleki et al., 2018), because of the aberrant efferocytosis in ADs (Kawane et al., 2010). In addition to creating a local transient inhibitory microenvironment, early apoptotic cells with intact cell membranes will ‘ask’ phagocytes to clear themselves by expressing specific signals (e.g. phosphatidylserine, PS) (Fadok et al., 1992), to prevent apoptotic cell explosion and the release of inflammatory factors called damage-associated molecular pattern (DAMP). Therefore, the inherent immunomodulatory properties of apoptotic cells have great potential to treat RA or other ADs (Poon et al., 2014; Toussirot et al., 2021). Bonnefoy et al. (2016) infused apoptotic thymocytes into CIA model, and the arthritis scores were significantly decreased by inducing the conversion of T cell and APC to a tolerogenic phenotype. Medina et al. (2020) tested that the metabolic mixture released by apoptotic cells dramatically reduced paw swelling and other arthritic parameters in RA mice, compared with treatment with vehicle controls. The factors in the supernatant of macrophages eliminating apoptotic cells also demonstrated anti-inflammatory effect on CIA model (Bonnefoy et al., 2018). In addition, PS-liposomes had some of the immunomodulatory characteristics of apoptotic cells compared to liposomes containing only phosphatidylcholine (Figure 4), and they could efficiently increase TGF-β1 secretion (Huynh et al., 2002).

Wu et al. (2010) found that blank PS-liposomes could halt inflammation in RA rats by raising plasma levels of TGF-β1 and prostaglandin E2, lowering the expression of NF-κB, blocking osteoclast differentiation and trabecular bone loss. Similar outcomes were confirmed in the work of H.M. Ma et al. (2011), PS-containing liposomes significantly reduce inflammatory bone loss in AIA rat. Even though this approach is antigen-independent, it has the potential to be a successful intervention to stop aberrant bone loss without having any negative side effects.

In addition, linking disease-related antigens to apoptotic cells can induce antigen-specific immune tolerance and enhance the tolerogenic effect produced by apoptotic cells alone (Getts et al., 2011). That typically resulted from the synergy of two mechanisms of action: activation of Tregs and programmed cell death 1 ligand 1 (PD-L1)-dependent T cell intrinsic unresponsiveness. However, technical complexity and costs associated with sourcing and peptide conjugation of donor cells are major obstacles to the clinical translation of this technique, and inert synthetic particles are expected to take the place of apoptotic cells’ usual role as carriers. Intravenous infusion of polystyrene or PLGA NPs (500 nm) coupled with encephalitogenic peptides using ethylene carbodiimide demonstrated to be an effective approach for inhibiting EAE, and the efficacy is superior to subcutaneous administration (Getts et al., 2012). To data, investigations on immunomodulatory nano-preparations mimicking apoptotic cells for the treatment of RA are uncommon.

Along with the discovery of T cell dominant epitopes specific to different ADs, urgent technological advances in the large-scale manufacture of synthetic particles as well as quality control are necessary when considering the clinical application of this technique.

## 6. Strategies based on scaffolds or gels

Scaffolds and gels made from macroscale biomaterials are three-dimensional polymeric networks that swell when exposed to water, aqueous medium, or physiological fluids. It has been widely employed to encapsulate and deliver cells, different medicinal compounds, and even drug-loaded nanoparticles to specific locations (Singh et al., 2009; Singh et al., 2011; Suri et al., 2011; Phelps et al., 2012; Verbeke et al., 2017; Zhao et al., 2021; Z. Li et al., 2022b). *In situ* vaccination of scaffolds and gels provides local niches and mimics the initiation of immune response during local infection (Adu-Berchie & Mooney, 2020). These substances recruit DCs and either stimulate or block their activation at the injection site, depending on the various immunomodulatory molecules (Ali et al., 2009; Verbeke et al., 2017), after which DCs with altered phenotypes migrate to secondary lymphoid organs to engage with T cells. Therefore, different from the systemic administration, NPs-loaded scaffolds or gels can be retained at the injection site to provide higher local self-antigen concentration (Stabler et al., 2019; Adu-Berchie & Mooney, 2020). Moreover, gel preparations are typically prepared via a modest crosslinking process, which is beneficial to preserve agent bioactivity (Singh & Peppas, 2014). In addition, scaffolds and gels endow the preparation with sustained- or controlled-release effects (Rambhia & Ma, 2015), and ongoing and stable antigen or adjuvant output promotes the development of the appropriate immune response (Demento et al., 2012; Awate et al., 2013; Zhou et al., 2016; Boggiatto et al., 2019). This tactic can increase effectiveness while minimizing systemic exposure and immune system effects.

Biomaterial scaffolds can rebuild immunological tolerance by enriching and modifying immune cells *in situ*. An injectable porous sodium alginate gel loaded with gold NPs coated with granulocyte-macrophage colony stimulating factor (GM-CSF) and PLGA microparticles coated with self-antigen was prepared (Verbeke et al., 2017). Animal studies demonstrated that intravenous injection efficiently established antigen-specific immunological tolerance in nonobese diabetic (NOD) mice, and up to 60% of antigen-specific CD4^+^T cells infiltrated into the gel are Tregs. F.X. Zhang et al. (2021) fabricated an AD/CS/RSF hydrogel with high adhesion strength to wet surface by cross-linking alginate dopamine (AD), chondriitin sulfate (CS), and regenerated silk fiber (RSF), in order to encapsulate exosomes (AD/CS/RSF/EXO) derived from bone marrow derived mesenchymal stem cells (BMSCs). Exosomes released from AD/CS/RSF/EXO recruited BMSCs into gel and neocartilage through chemokine signaling pathway after implantation in osteoarthritis rats, significantly promoting cartilage defect regeneration and extracellular matrix remodeling *in situ*.

DMARDs and GCs are now the main focus of investigations using NPs-loaded scaffolds and gels for RA, while reports of antigen-specific immunotherapy based on this approach are scarce.

## 7. Discussion

Inducing and maintaining robust immunological tolerance has been the holy grail of immunotherapy for decades (Bluestone & Anderson, 2020). However, different from the effective outcomes in animal experiments, clinical translation of tolerogenic nano-preparation is exceedingly complicating and vexing. Several reasons may have resulted in this phenomenon, including: (1) The animal model of RA cannot accurately imitate the pathophysiological mechanisms of human RA. For instance, the commonly used murine RA models are an acute pathological process induced by a single injection of CFA or two injections of CFA with specific antigens, while real RA is a chronic disease with numerous causes; (2) Exact identification of the main or dominant self-antigens responsible for human RA is lacking. (3) The treatment options are vastly dissimilar. Treatment in animal experiments frequently begins before the development of a model or at the commencement of symptoms, whereas treatment in clinical trials typically begins after the onset of disease; (4) There are significant physiological and immune system differences between humans and murine, which may cause different distribution and immune response of immunomodulatory nano-preparation in different species; (5) The polymorphism of human MHC alleles (Radwan et al., 2020; Abualrous et al., 2021) may affect the dominant epitopes that are active in different RA individuals (Okada et al., 2014).

Fortunately, with the rapid development of immunology, pharmacy, materials science, etc., animal models, neoantigens, and delivery vectors that are more suitable for studying immunotherapy of RA will emerge. Humanized RA mouse models such as human RA transgenic mice and murine–human chimera models have been currently used in preclinical investigations (Schinnerling et al., 2019). Efforts are underway to develop techniques for predicting the onset of RA and T_1_D (Raychaudhuri et al., 2017; Yi et al., 2018; Harms et al., 2020; Su, 2020). Moreover, RA therapies based on the cellular delivery systems are on the rise (Q. Zhang et al., 2018; Q. Ma et al., 2021), and the application of myelin whole antigen is overcoming the barrier to clinical translation of MS animal models (Casella et al., 2020). In addition, the widespread vaccination of Comirnaty and Spikevax for coronavirus disease 2019 (COVID-19) prevention in the population further verifies the safety and effectiveness of LNP technology (Thompson et al., 2021). We anticipate that the platform technology of immunomodulatory nano-preparation will soon be successfully applied to treat RA, MS, T_1_D, and other ADs with the help of those cutting-edge technologies.
